# Evaluation of children's antibiotics use for outpatient pneumonia treatment in Vietnam

**DOI:** 10.1016/j.bjid.2024.103839

**Published:** 2024-07-09

**Authors:** Tuong Vi Le Thi, Em Canh Pham, Doan-Trang Dang-Nguyen

**Affiliations:** a University of Medicine and Pharmacy at Ho Chi Minh City, Faculty of Pharmacy, Ho Chi Minh City, Viet Nam; b City Children's Hospital, Faculty of Pharmacy, Department of Pharmacology - Clinical Pharmacy, Ho Chi Minh City, Viet Nam; c Hong Bang International University, Faculty of Pharmacy, Ho Chi Minh City, Viet Nam

**Keywords:** Antibiotic, Outpatient pneumonia, Children, Prescription, CAP

## Abstract

**Objective:**

Antibiotic resistance is increasing globally, associated with many failures in pneumonia treatment. This study aimed to evaluate antibiotic use in children treated for outpatient CAP (Community-Acquired Pneumonia).

**Methods:**

A cross-sectional descriptive retrospective study was conducted, focusing on data from outpatient prescriptions for pneumonia in patients aged 2‒192 months in 2019‒2021.

**Results:**

All antibiotic prescriptions are considered empiric as no documented bacterial and viral tests exist for children with non-severe CAP. Single antibiotic therapy (66%) had a 2-fold higher rate than combination therapy (34%). Amoxicillin/clavulanic acid (50.77%) and azithromycin (30.74%) were the most commonly prescribed in both single and combination therapies, thus determining antibiotic cost (80.15%). Besides, azithromycin (97.92%), cefuroxime (86.26%), and cefpodoxime (60.48%) were prescribed with high adherence to dose guidelines, except for amoxicillin (34.57%). These medicines are prescribed highly compliant (>83%) with dosing interval guidelines. Furthermore, significantly more brand-name antibiotics (56.5%) are prescribed than generic antibiotics (43.5%). In particular, antibiotic class, antibiotic origin, and antibiotic therapies showed significant association with rational antibiotic prescriptions for dose and dose interval (p < 0.05).

**Conclusions:**

Amoxicillin/clavulanic acid is the most frequently prescribed medicine and the most inappropriate due to non-compliance with dose guidelines for CAP treatment. Generic antibiotic use for single therapy should be encouraged based on rapid and accurate diagnostic testing for viruses and bacteria to reduce antibiotic resistance in developing countries. Moreover, the study result has also shown that therapies and antibiotics (class and origin) exhibited significant association with rational prescriptions for CAP treatment for pediatrics.

## Introduction

Community-Acquired Pneumonia (CAP) is one of the most common infectious diseases and remains an important cause of morbidity, high rates of hospitalization, and mortality in children worldwide, especially in children <5 years-old (accounts for 15% of all deaths).^1^ The empiric antibiotic therapy based on clinical presentation is often used for CAP because the clinical presentation of CAP is often non-specific in young children, and bacterial and viral pneumonia may be clinically indistinguishable.^2^ Limiting exposure to any antibiotic and identifying the causative organism is very difficult to achieve with antibiotic treatment selects for antibiotic resistance.^2^ An incidence of 0.29 episodes per child per year and more than 2 million children aged 0 to 5-years die (or a mortality rate of 1.3%‒2.6%) from pneumonia each year, accounting for nearly one-fifth of all deaths among children under the age of five worldwide.^3^ However, its incidence and mortality rate is significantly higher in developing countries than in the industrialized world (an incidence of 0.05 episodes per child per year). This is likely due to a disproportionate and multifactorial influence on the incidence of CAP in children in developing countries, including vaccine implementation, access to healthcare, nutrition, and living conditions.

Appropriate antibiotic treatment is the main weapon to reduce mortality in children with bacterial pneumonia. In contrast, inappropriate antibiotic use (like overuse of antibiotics, using the wrong dose, using the wrong time, using the wrong combination of antibiotics) contributes to antibiotic resistance, causing many side effects, reducing the effectiveness of the treatment of infections, increasing healthcare costs, and raising the burden on the National Health System.^4^^,^^5^ Besides, the overuse of antibiotics has become a leading health concern in many countries. According to statistics from the European Medicines Agency, it is estimated that there are about 25,000 deaths yearly due to multidrug-resistant bacterial infections and the economic burden of antibiotic resistance is up to €1.5 billion per year.^6^ In addition, WHO has warned that antibiotic resistance is a major threat to the ability to treat lung infections in the community and in hospitals, causing treatment failure, prolonging hospital stays, and the threat to the patient's life. Antibiotic resistance is predicted to be the cause of approximately 10 million deaths annually by 2050 and cost over $100 trillion worldwide.^7^ Therefore, promoting rational antibiotic prescribing is a global priority, especially in Asia, where pneumonia in children is a major driver of antibiotic use and access to antibiotics is largely unrestricted.^4^^,^^8^ In addition, recommendations for the use of narrow-spectrum antibiotics as first-line treatment for CAP in both outpatients and hospitalized patients for children over three months of age are reflected in current Vietnamese and international guidelines.^9^ So, guidelines typically recommend oral amoxicillin as the first-line treatment for non-severe pneumonia.^10^^,^^11^ Moreover, non-severe pneumonia can be managed as an outpatient with a 5-day course of oral antibiotics.^12^ Despite international recommendations and published evidence supporting this approach, broad-spectrum antibiotics continue to be over-prescribed, and prolonged treatment times create a large financial burden on families.^13^

The analysis of the current antibiotic use situation is necessary for doctors and managers to develop and implement a strategy for the safe and rational use of antibiotics, which is also a solution to improve the efficiency of the treatment of CAP in children. However, there is a paucity of reports from Vietnam tertiary hospitals on pediatric outpatient prescribing and the rationality of antibiotic prescriptions for CAP. Therefore, the aim of the present study is to review the outpatient prescription status and evaluate the rationality of antibiotic use to treat CAP for children in Vietnam.

## Materials and methods

### Study center

City Children's Hospital (Vietnam) is one of the most extensive tertiary pediatric hospitals under the Department of Health of Ho Chi Minh City with a 1000‑bed large capacity in Southern Vietnam.

### Study design

The present study used a cross-sectional descriptive retrospective method with a non-clinical approach based on CAP-treated outpatient prescriptions.

### Clinical criteria

The diagnosis of CAP is based on the presence of respiratory symptoms and a chest X-Ray with the appearance of new lung opacity or peripheral blood test or C-reactive protein analysis. Non-severe pneumonia is diagnosed in a child with cough or difficulty in breathing, with rapid breathing (a respiratory rate of at least 30 breaths per min in a child aged ≥ 60-months, or at least 40 breaths per min in a child aged 12‒59 months, or at least 50 breaths per min in an infant aged 2‒12 months).

### Inclusion criteria

All pediatric patients of both genders, aged ≥2‒192 months, attended outpatient treatment between January 2019 and December 2021 with the client's hospital number and at least one prescribed antibiotic for CAP with or without other medications. This approach was adopted to obtain a complete spread over the study period and to minimize bias due to seasonal variations in disease expression and disruption of drug supply cycles.

### Exclusion criteria

Non-antibiotic prescriptions for CAP without hospital numbers and the stipulated age and inpatient prescriptions were excluded from this study.

### Data collection

The information on eligible prescriptions was filtered from Hospital Information Software (HIS 4.0, VNPT ‒ Vietnam Posts and Telecommunications Group) from pharmacies at City Children's Hospital, Vietnam. The clinical pharmacists gathering data underwent intensive observation and training. In addition, the accuracy of the data collected was ensured by repeated and cross-reviewed by five clinical pharmacists.

The study's dependent variable was the antibiotic prescribing status and rationality for CAP treatment. The independent variables were the sociodemographic factors (including client's hospital number, full name, date of birth, body weight gender, age, place of residence, date of admission, and diagnosis) and antibiotic knowledge (like name, dose, route, content, frequency, dosage form, number of antibiotic drugs prescribed, number of uses, unit price, and duration of treatment).

### Data analysis

Data collected were transferred into SPSS 26 software (IBM Corporation, Armonk, NY), which was used for data analysis. Qualitative variables were reported using frequency counts and percentages. Meanwhile, quantitative data were calculated using descriptive statistics and presented as mean and according to the type of distribution of each variable.

The difference between the two groups was analyzed by one-way analysis of variance (ANOVA) with Tukey's Honestly Significant Difference (Tukey HSD) post hoc test. The association of demographics and clinical characteristics for outpatient CAP treatment was analyzed using the χ2 test. The results were considered statistically significant if p < 0.05. The chart is drawn using Microsoft Excel 2022 software.

### Rationality of antibiotic use

In the present study, prescriptions are considered rational when antibiotics are prescribed according to the correct regimen including indications, route of administration, dosage, frequency of administration, and duration of treatment. Antibiotic dosages and dosing intervals were compared with the guideline for diagnosing and treating pediatrics of the American Academy of Pediatrics and WHO (Table 1).^10^^,^^11^

**Table 1:** Dose criteria for the assessment of the reasonableness of CAP ‒ treated outpatient prescription.

Antibiotic	Dose (mg/kg/day)	Dose interval (time/day)	Ref
Amoxicillin	90	2	^10^
Amoxicillin/Clavulanic acid	80‒90	2	^10^
Amoxicillin/Sulbactam	100	2	*
Cefuroxime	20‒30	2	^10^
Cefaclor	20‒40	2‒3	^10^
Cefdinir	14	1‒2	^10^
Cefditoren	9	3	*
Cefixime	8	1‒2	^10^
Cefpodoxime	10	2	^10^
Azithromycin	5‒10	1	^10^
Clarithromycin	15	2	^10^
Erythromycin	40‒50	3-4	^10^
Clindamycin	10‒25	3	^10^
Ciprofloxacin	20‒40	2	^10^
Levofloxacin	16‒20^a^	2^a^	^11^
	10^b^	1^b^	

a Aged 6-months to 5-years.

b Older than 5-years.

⁎ According to the drug leaflet.

### Ethical consideration

Ethical approval to conduct the research was obtained from City Children's Hospital (Vietnam) Health Research Ethics Committee on February 17, 2022, with n° CS/NĐTP/22/07.

## Results

### Pediatric patient characteristics for outpatient CAP therapy

A total of 398,798 records of children were retrieved and screened. The total number of outpatient prescriptions using antibiotics for pneumonia in children (aged ≥ 2-months) during the 3-years of the retrospective cross-sectional study was 3,555 (Supplementary Table S1 and Fig. S1). The mean-month-old of CAP children was 27.73±24.02 with the lowest and highest months-old being 2 and 172.63, respectively (Supplementary Table S2). The percentage of boys (54.7%, n = 1,944) and girls (45.3%, n = 1,611) exhibited significant differences (p < 0.05). In addition, the percentage of children from 2 to <60 months old who received outpatient CAP treatment was the highest at 92.6% (n = 3,292). This group showed a significant difference (p < 0.05) compared to the children group of ≥ 60-months old (7.4%, n = 263).

### The antibiotic prescribing status for CAP

**Fig. 1:**
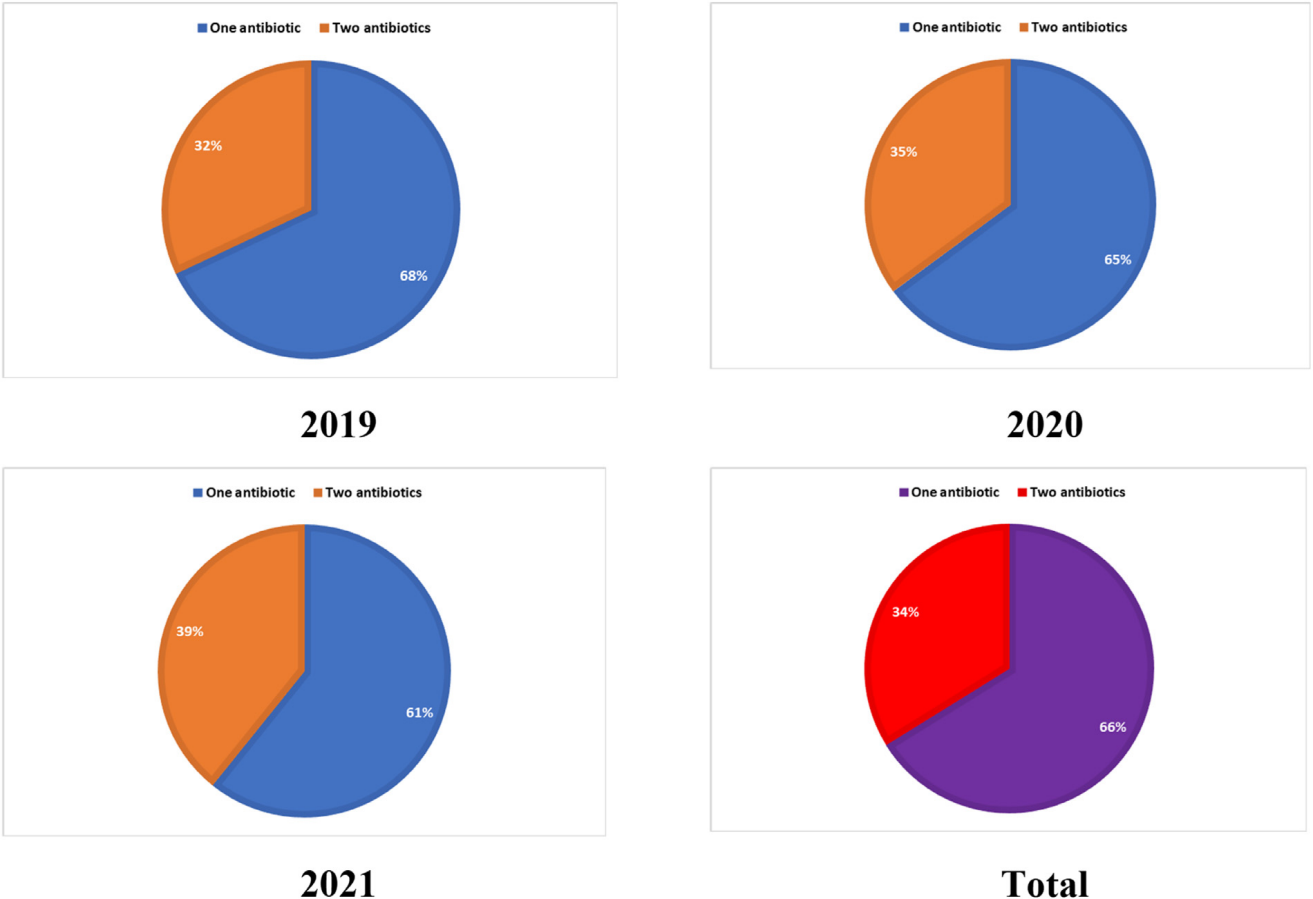
Rate of single antibiotic and combination therapy in the outpatient treatment of community-acquired pneumonia in children.

All antibiotics contained in the outpatient CAP prescription orders were available in the hospital pharmacy. The average number of antibiotics prescribed per encounter for outpatient CAP treatment was 1.34, and the average antibiotics treatment was about 3 days. The mean percentage of antibiotic-using outpatient prescriptions for CAP treatment was 0.9% compared to all outpatient prescriptions in three years. The majority of prescriptions (66%, n = 2,352) had single antibiotics, and two antibiotics combination was administered in 34% (n = 1,203) (Fig. 1).

**Fig. 2:**
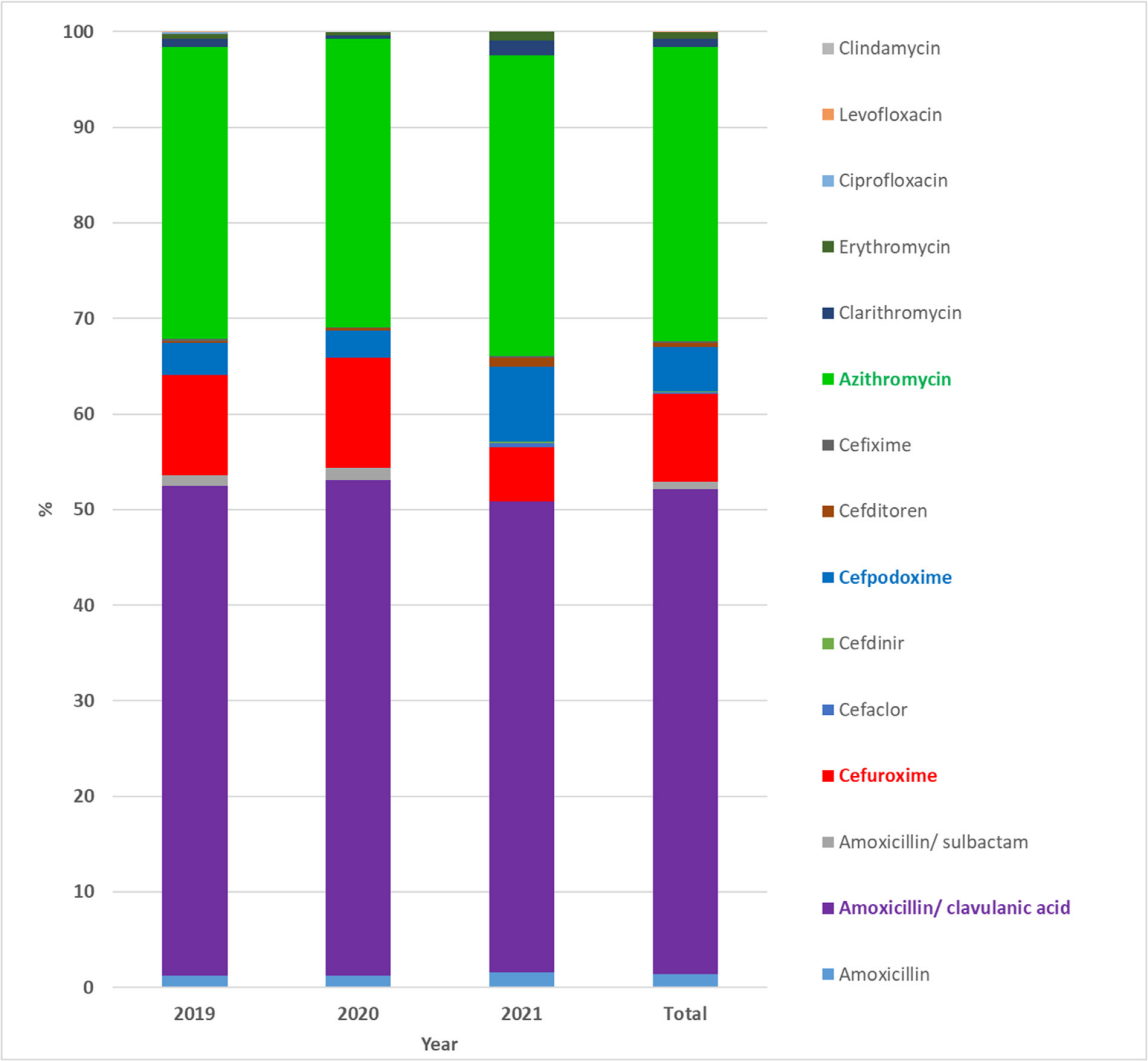
Prescription proportion (%) of each antibiotic for CAP treatment (Total ‒ average of 3years).

Among the classes of antibiotics, *β*-lactams (67.65%, including 52.96% penicillin and 14.69% cephalosporin) and macrolides (32.28%) were prescribed the most frequently for outpatient CAP treatment. The 2^nd^ and 3^rd^ generation cephalosporin antibiotic groups were used in percentages of 9.35% and 5.34%, respectively (Table 2). On the other hand, amoxicillin/clavulanic acid is the most commonly prescribed antibiotic and accounted for 50.77% of prescriptions for the first choice at the age of 2-months to 5-years (Fig. 2). Besides, azithromycin (30.74%), cefuroxime (9.18%), and cefpodoxime (4.68%) antibiotics were prescribed alone or in combination with other antibiotics.

**Table 2:** Overall usage of outpatient antibiotics among children with CAP.

Antibiotic		2019	2020	2021	Total^a^		
Group	Name	n (%)	n (%)	n (%)	%	%	%
Penicillin	Amoxicillin	34 (1.26)	18 (1.27)	10 (1.56)	1.37	1.37	**52.96**
Penicillin/*β*-lactamase inhibitor	**Amoxicillin/Clavulanic acid**	1387 (**51.24**)	731 (**51.77**)	315 (**49.30**)	**50.77**	**51.59**	
	Amoxicillin/Sulbactam	30 (1.12)	19 (1.35)	0 (0.00)	0.82		
2^nd^ generation Cephalosporin	**Cefuroxime**	282 (**10.44**)	162 (**11.47**)	36 (**5.63)**	**9.18**	**9.35**	**14.69**
	Cefaclor	2 (0.05)	0 (0.00)	3 (0.47)	0.17		
3^rd^ generation Cephalosporin	Cefdinir	0 (0.00)	0 (0.00)	1 (0.16)	0.05	**5.34**	
	**Cefpodoxime**	90 (**3.32**)	41 (**2.90**)	50 (**7.82**)	**4.68**		
	Cefditoren	5 (0.20)	3 (0.21)	6 (0.94)	0.45		
	Cefixime	7 (0.24)	1 (0.07)	1 (0.16)	0.16		
Macrolide	**Azithromycin**	826 (**30.52**)	427 (**30.24**)	201 (**31.46**)	**30.74**	32.28	**32.28**
	Clarithromycin	22 (0.83)	5 (0.35)	10 (1.56)	0.91		
	Erythromycin	16 (0.59)	5 (0.35)	6 (0.94)	0.63		
Fluoroquinolone	Ciprofloxacin	4 (0.15)	0 (0.00)	0 (0.00)	0.05	0.07	0.07
	Levofloxacin	2 (0.05)	0 (0.00)	0 (0.00)	0.02		
Lincosamide	Clindamycin	0 (0.00)	0 (0.00)	0 (0.00)	0.00	0.00	0.00
**Total**		2707 (100)	1412 (100)	639 (100)	100	100	100

n, Frequency (number of times/year).

a Average of 3-years.

**Fig. 3:**
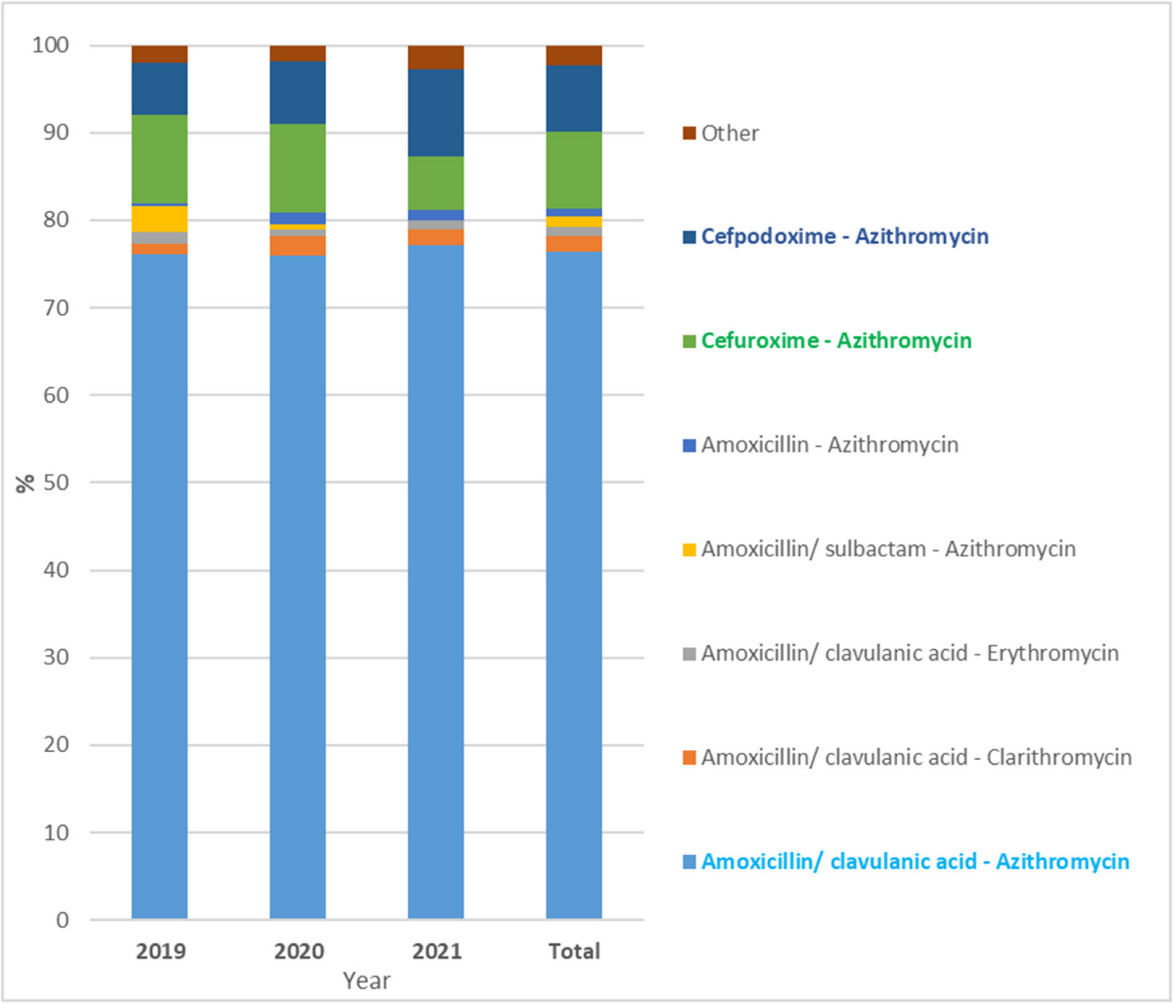
Prescription proportion (%) of antibiotic combinations for CAP treatment (Total ‒ average of 3-years).

Among the 1,203 prescriptions with two antibiotic combination therapy, 100% of prescriptions were followed by *β*-lactam antibiotics combined with macrolide antibiotics. The combination of penicillin ‒ macrolides antibiotics was used the most with a percentage of 81.30%, and the cephalosporin ‒ macrolides antibiotics combination was used with a percentage of 18.70%. Specifically, amoxicillin/clavulanic acid ‒ azithromycin (76.44%) was the most common antibiotic combination for outpatient CAP treatment in Vietnamese children (Fig. 3 and Supplementary Table S3). In addition, cefuroxime ‒ azithromycin (8.80%) and cefpodoxime ‒ azithromycin (7.68%) was commonly used in the order 2^nd^ and 3^rd^, respectively.

### The rationality of antibiotic use for outpatient CAP treatment

**Fig. 4:**
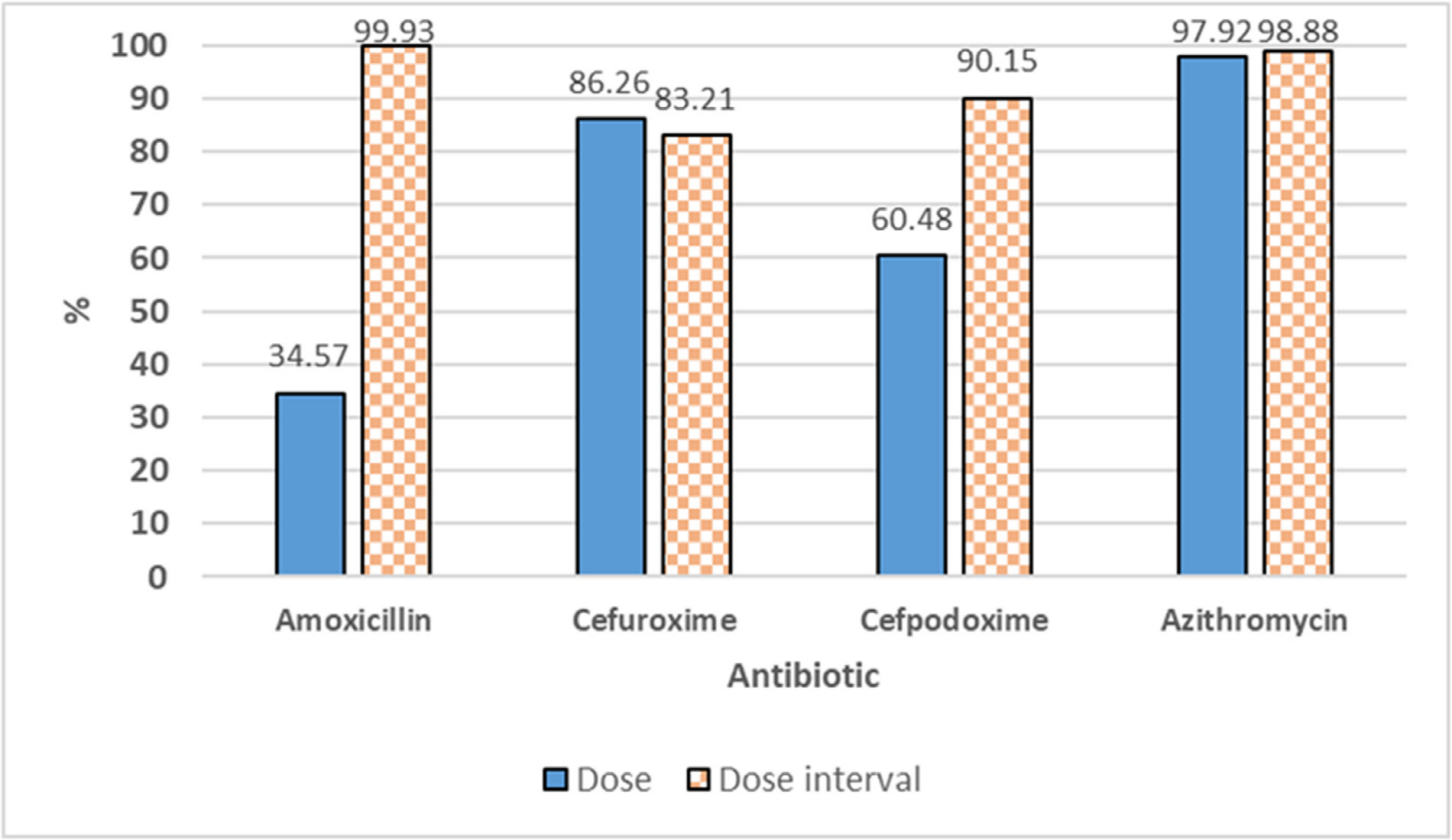
Comparison of guideline adherence for common antibiotic prescriptions.

All prescriptions demonstrated appropriate antibiotic selection for CAP treatment according to disease status and age with the guidance of the Ministry of Health of Vietnam. However, amoxicillin/clavulanic acid is the most prescribed antibiotic whereas WHO guidelines recommended amoxicillin alone. Cases of antibiotic combinations have only been prescribed in CAP children who did not respond to prior single-antibiotic therapy with non-severe pneumonia symptoms showing no signs of remission, but antibiotic combination for non-severe pneumonia is not recommended by WHO. Moreover, the majority (98.45%) of prescriptions were appropriate regarding indications, administration route, and treatment duration. Therefore, the rational antibiotic prescription was assessed primarily by dose and dose interval. Guideline non-adherence per antibiotic is presented in Table 3. Analysis revealed that azithromycin, cefuroxime, and cefpodoxime as popularly prescribed antibiotics showed a high adherence to the dose guidelines with mean percentages of 97.92%, 86.26%, and 60.48%, respectively. However, amoxicillin as the most popular prescription antibiotic (>50%) showed moderate adherence to the dose guidelines with a mean percentage of 34.57%, of which amoxicillin/clavulanic acid made up the largest proportion of guideline-noncompliant prescriptions. In addition, all four commonly prescribed antibiotics exhibited high dose interval compliance with the mean percentages of amoxicillin, azithromycin, cefuroxime, and cefpodoxime being 99.93%, 98.88%, 83.21%, and 90.15%, respectively (Fig. 4). Besides, adherence to the dose guidelines of lactam, macrolide, and other antibiotic groups was 45.1%, 96.3% and 66.7%, respectively. The adherence to the dose interval guidelines of β-lactam, macrolide, and other antibiotic groups was 96.4%, 97.4%, and 100%, respectively. Calculating in the prescription unit, dose and dose interval adherence were 38.4% (n = 1,365) and 94.8% (n = 3,370), respectively (Supplementary Fig. S2).

**Table 3:** Adherence with antibiotic prescribing guidelines for CAP treatment.

Antibiotic	2019		2020		2021		Total^b^	
	Dose	Dose interval	Dose	Dose interval	Dose	Dose interval	Dose	Dose interval
	n (%)	n (%)	n (%)	n (%)	n (%)	n (%)	%	%
Amoxicillin^a^	570 (39.28)	1450 (99.93)	268 (34.90)	767 (99.87)	96 (29.54)	325 (100.00)	34.57	99.93
Cefuroxime	231 (81.91)	227 (80.50)	138 (85.19)	130 (80.25)	33 (91.67)	32 (88.89)	86.26	83.21
Cefaclor	2 (100.00)	2 (100.00)	‒	‒	3 (100.00)	3 (100.00)	100.00	100.00
Cefdinir	‒	‒	‒	‒	0 (0.00)	0 (0.00)	‒	‒
Cefditoren	0 (0.00)	4 (80.00)	1 (33.33)	2 (66.67)	3 (50.00)	6 (100.00)	27.78	82.22
Cefixime	2 (28.57)	6 (85.71)	1 (100.00)	1 (100.00)	0 (0.00)	1 (100.00)	42.86	95.24
Cefpodoxime	52 (57.78)	74 (82.22)	22 (53.66)	37 (90.24)	35 (70.00)	49 (98.00)	60.48	90.15
Azithromycin	802 (97.09)	814 (98.55)	417 (97.66)	421 (98.59)	199 (99.00)	200 (99.50)	97.92	98.88
Clarithromycin	13 (59.09)	22 (100.00)	3 (60.00)	5 (100.00)	4 (40.00)	9 (90.00)	53.03	96.67
Erythromycin	13 (81.25)	4 (25.00)	5 (100.00)	1 (20.00)	6 (100.00)	2 (33.33)	93.75	26.11
Ciprofloxacin	4 (100.00)	4 (100.00)	‒	‒	‒	‒	100.00	100.00
Levofloxacin	0 (0.00)	2 (100.00)	‒	‒	‒	‒	0.00	100.00
Clindamycin	‒	‒	‒	‒	‒	‒	‒	‒

n, Frequency (number of times per year of appropriate prescriptions according to the guidelines).

a Amoxicillin or Amoxicillin/Clavulanic acid or Amoxicillin/Sulbactam.

b Average of 3-years.

The association of demographics and clinical characteristics for outpatient CAP treatment was shown in Table 4. There was a statistically significant association between the origin of antibiotics (generic and brand-name) with age and place of residence as well as between antibiotic class (lactam, macrolide, and other) with age with p-values<0.05. In contrast, there was no statistically significant association between gender with antibiotic treatment, types of drug substance, and origin of medicine with p-values of 0.116, 0.793, and 0.622 respectively. Age showed an association with rational and irrational antibiotic prescriptions regarding dose interval (p-value of 0.047) but not regarding dose (p-value of 0.438). There was no statistically significant association between gender and place of residence with dose and dose interval guideline adherence with p-values>0.05. Furthermore, antibiotic treatment (one and two antibiotics), antibiotic class, and antibiotic origin exhibited significant association with rational and irrational antibiotic prescriptions (dose and dose interval) with p-values < 0.05.

**Table 4:** The association of demographics and clinical characteristics for outpatient CAP treatment.

		Antibiotic treatment		p-value^d^	Drug substance			p-value^d^	Origin of medicine		p-value^d^
Characteristics		One	Two		Lactam	Macrolide	Other		Generic	Brand-name	
**Gender**	Male	1307	634	0.116	1759	817	3	0.793	1250	1329	0.622
	Female	1045	569		1475	701	3		1040	1138	
**Age**^a^	2 to <60	2213	1075	0.238	3017	1347	5	**0.000**	2055	2314	**0.000**
	≥60	139	128		217	171	1		234	154	
**Place of residence**	City^b^	1172	502	0.714	1518	665	2	0.113	1139	1047	**0.000**
	Other	1180	701		1716	853	4		1152	1421	
		Dose		p-value	Dose interval		p-value	Total		p-value	
		0	1		0	1		0	1		
**Gender**	Male	1207	737	0.371	106	1838	0.284	1254	690	0.334	
	Female	976	634		75	1535		1014	597		
**Age**^a^	2 to <60	2007	1285	**0.047**	165	3127	0.438	2085	1208	**0.034**	
	≥60	176	86		16	246		183	79		
**Place of residence**	City^b^	1055	626	0.121	84	1597	0.806	1090	591	0.220	
	Other	1128	745		97	1776		1178	696		
**Antibiotic treatment**	One	1385	967	**0.000**	104	2248	**0.004**	1439	913	**0.000**	
	Two	805	398		80	1123		837	366		
**Drug substance**^c^	Lactam	1777	1457	**0.000**	118	3116	**0.007**	‒	‒		
	Macrolide	56	1462		40	1478		‒	‒		
	Other	2	4		0	6		‒	‒		
**Origin of medicine**	Generic	974	692	**0.001**	133	1533	**0.000**	1032	635	**0.028**	
	Brand-name	1209	679		48	1840		1236	652		

One, Single antibiotics; Two, Two antibiotics.

a Aged month, 0- Guideline non-adherence, 1- Guideline adherence, Total- Compliance - agreed on both dose and dose interval guidelines for all antibiotics in each prescription.

b Ho Chi Minh City.

c Calculated in antibiotic units.

d Using the χ2 test.

### Cost of antibiotics for outpatient CAP treatment

**Fig. 5:**
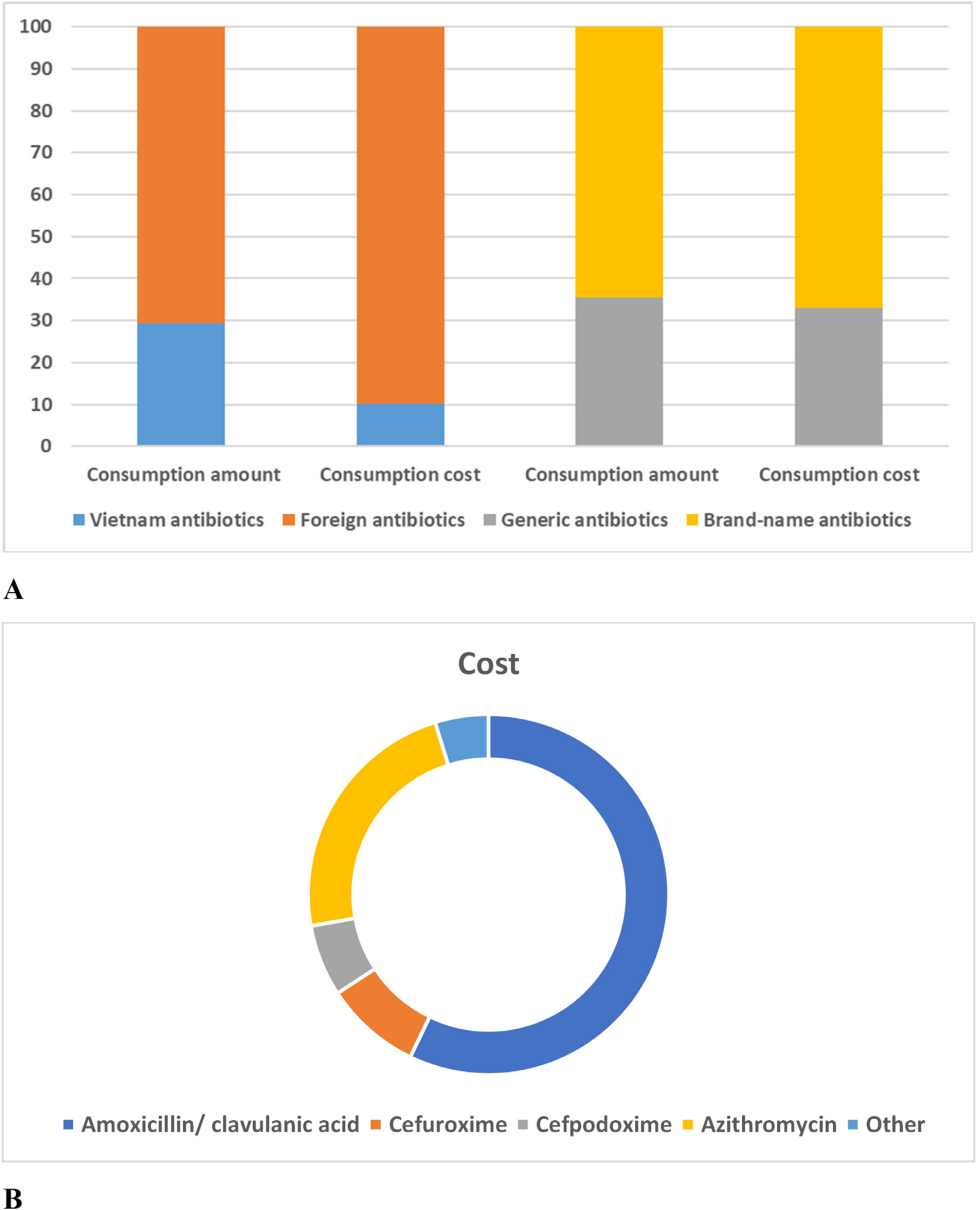
Comparison of antibiotic costs for outpatient CAP treatment (A, Cost by the origin of antibiotics; B, Cost by antibiotic class).

The percentage of outpatient treatment costs for CAP children by origin and antibiotic class are summarized in Fig. 5. Children with mild pneumonia were given oral antibiotics with an average cost of approximately US$1.0 per prescription, accounting for 2.5% of total hospital-wide antibiotic costs. The consumption amount of imported antibiotics (70.78%) has been shown to be about 2.5 times higher than that of Vietnam antibiotics (29.21%). Besides, the consumption cost of foreign antibiotics (89.79%) has been shown to be about 9.0 times higher than that of Vietnam antibiotics (10.21%). On the other hand, the consumption amount and cost of brand-name antibiotics (65.79%) were shown to be about 2.0 times higher than that of generic antibiotics (34.21%) (Supplementary Table S4).

For the consumption cost of each antibiotic, the prescribing cost for amoxicillin antibiotics accounted for 58.17%. The cost of amoxicillin/clavulanic acid (57.15%) accounted for the highest percentage compared to other antibiotics prescribed for outpatient CAP at the Vietnam hospital (Fig. 5 and Supplementary Table S5). In addition, the three antibiotics azithromycin, cefuroxime, and cefpodoxime also accounted for a large share of the prescribing costs for pneumonia with 23.00%, 8.64%, and 6.42%, respectively. The cost for the remaining antibiotics accounted for less than 2.5% per antibiotic.

## Discussion

The predominant (> 92%) in the 2-month-5-year-old group was found to be similar to many published studies and depicts the higher chances for inappropriate prescribing for younger children exposing them to risks of side effects.^14^, ^15^, ^16^, ^17^ In addition, a male predominance was found in outpatient CAP treatment with a male and female ratio of 1.2:1, similar to some studies.^15^^,^^18^ Furthermore, more than 2,000 pediatric patients (> 50%) were admitted to the general internal ward. Besides, 80.59% (n = 2,865) of children only had pneumonia on outpatient prescriptions and 19.41% (n = 690) of children had recorded pneumonia with other diseases like fever (4.39%, n = 156), asthma (4.14%, n = 147), measles (2.05%, n = 73), upper respiratory tract infection (2.11%, n = 75), and digestive disorders (3.94%, n = 140). However, fever or digestive disorders could be explained by CAP.

The majority (> 50%) of pediatric patients tried home medication before visiting a hospital, especially children living outside Ho Chi Minh City (the largest city in Vietnam). The practice of antibiotic self-medication is a known risk factor for the development of antibiotic resistance.^19^ This finding is similar to some studies in India and Nigeria with the rate of CAP children of home-based treatment being 80.7% and 75.2%, respectively.^20^^,^^21^ In addition, inappropriate antibiotic use is associated with easy access due to the lack of adequate drug regulatory mechanisms in most countries.^22^

The majority of pediatric patients fulfill WHO pneumonia criteria, which were supported by a chest radiograph (63.8%), peripheral blood test (33.6%), and C-reactive protein (27.4%) analysis. The majority (74.4%) of cases where antibiotics were prescribed ordered lab investigation for “non-severe” CAP pediatric patients such as chest radiograph, peripheral blood test, and C-reactive protein analysis. Whereas 24.6% of cases were prescribed antibiotics without any lab investigation. Moreover, no pediatric patients are indicated for simple biomarker tests like procalcitonin, cultures, and viral rapid tests to distinguish between viral and bacterial infections, especially in non-severe cases. Therefore, the rate of prescriptions diagnosed with pneumonia with an unknown agent was high (61.5%, n = 2,186). The study results showed that antibiotics were prescribed for outpatient treatment of pneumonia without any confirmed bacterial infection. Before the age of 5-years old, a significant proportion of infections had a viral origin and would not require antibiotics. Although respiratory viruses are the most commonly isolated respiratory pathogens, many doctors are uncomfortable excluding bacterial pneumonia. This may be due to limited experience with WHO pneumonia management guidelines and low rates of pneumococcal vaccination in Vietnam. The diagnostic value of biomarkers and rapid viral tests has limitations, but these tests can help increase appropriate antibiotic use,^9^ whether to be de-escalated, broadened, stopped, or identify viral etiology in some cases.^16^^,^^23^ Therefore, the designation of rapid viral testing for CAP children should be encouraged to be carried out regularly in hospitals to reduce antimicrobial use in children with non-severe viral CAP.

An increase in antibiotic use in pediatric patients has been found in the outpatient treatment of CAP in Vietnam, similar to that in Italy,^16^^,^^24^ UK,^24^ Netherlands,^24^ India,^15^ Nigeria,^14^ and Saudi Arabia.^25^ Two-thirds (> 60%) of CAP prescriptions contained single antibiotics, similar to that reported in Nigeria (71.4%).^26^ In the single antibiotics prescriptions, amoxicillin contributed the highest percentage share at 66.5% (n = 1,564/2,352) followed by cefuroxime (15.1%, n = 354/2,352), azithromycin (12.8%, n = 300/2,352), and cefpodoxime (4.0%, n = 95/2,352). The *β*-lactam antibiotics were the most prescribed, accounting for two-thirds of antibiotic prescriptions for outpatient CAP in Vietnam children, especially amoxicillin/ clavulanic acid accounting for about 50% of antibiotic prescriptions. In addition to the broad antimicrobial spectrum, antibiotic costs are also considered in prescribing alternative antibiotics and antibiotic combinations on the essential medicines list in Vietnam. Choosing an oral antibiotic from a system-available formulary reduces costs and the potential for infection if applicable.^27^ Most guidelines recommend the use of amoxicillin as the first-line antibiotic in non-severe pneumonia unless the patient has an allergy.^10^^,^^12^ It is possible that clinicians are more cautious and prone to prescribe broad-spectrum antimicrobials such as amoxicillin/ clavulanic acid because of the inability to observe the clinical outcome of patients on a daily basis.^28^ Besides, penicillins, particularly amoxicillin, are cheap, covered by health insurance, and readily available over the counter from community drug outlets. Moreover, high-dose amoxicillin remains an excellent antibiotic choice in pediatric pneumonia cases because *Streptococcus pneumoniae* isolated from respiratory tract specimens is highly susceptible to penicillins and lowly susceptible to cephalosporins and macrolides in Vietnam.^29^ These are the main reasons explaining the finding of oral amoxicillin/clavulanic acid as the most common antibiotic from which the prescriptions were made.^26^ Most published studies have also shown amoxicillin to be the most commonly prescribed antibiotic and the use of broad-spectrum *β*-lactams is increasing.^18^

Azithromycin (macrolide) antibiotic is also highly prescribed in about one-third of all outpatient prescriptions, similar to some other studies.^30^ Meanwhile, one-sixth of outpatient CAP prescriptions contained 2^nd^ and 3^rd^ generation cephalosporin antibiotics. Cefuroxime (2^nd^ generation cephalosporin) and cefpodoxime (3^rd^ generation cephalosporin) were often prescribed in this antibiotic class as standard practice without any disease severity or microbiological consideration. Unfortunately, the use of oral third-generation cephalosporins has shown an upward trend in outpatient treatment in Vietnam, similar to other Asian regions where its use is widespread.^29^^,^^31^ Although there is no proven benefit in CAP children, most doctors generally assume that most children have received antibiotics before going to the hospital and prefer to use broad-spectrum antibiotics.

Moreover, one-third of CAP prescriptions contained two antibiotics, suggestive of a high rate of combination therapy to broaden the spectrum of antimicrobial action. The most common antibiotic combination is amoxicillin/clavulanic acid and azithromycin (76.44% of prescriptions for two antibiotics). The cefuroxime ‒ azithromycin (8.80%) and cefpodoxime ‒ azithromycin (7.68%) combinations are frequently used similarly in other Asian countries.^13^ These combinations help to extend the antibacterial spectrum to include both typical and atypical bacteria organisms such as *Streptococcus pneumoniae, Haemophilus influenzae* type b, and *Mycoplasma pneumoniae*, which are widely considered to be the leading cause of CAP. However, high rates of macrolide resistance recorded in multiple settings suggest futility and unnecessary costs.^8^ Unfortunately, combination prescriptions have shown an increasing trend in prescribing for CAP in the present study. This means using more antibiotics in a prescription, which can lead to antibiotic resistance, increased side effects, increased risk of harmful drug interactions, dispensing errors, non-adherence to medications, and increased financial costs for families.

On the other hand, the adherence to dose interval prescribing guidelines for antibiotics was found to be higher than for dose in the present study. Amoxicillin/clavulanic acid showed the largest proportion (> 60%) of non-compliance with dose guidelines, emphasizing the need for information on antibiotic use per clinical care setting to direct Antibiotic Stewardship Programs.^32^ Most children were treated with doses of amoxicillin antibiotic lower than those recommended by national and international guidelines. In Australia, above 50.0% of antibiotic prescriptions were contrary to the guidelines.^33^ Similar to this study, a high rate of noncompliance in antibiotic prescribing in the outpatient setting has also been demonstrated in many previous studies for respiratory tract infections.^34^^,^^35^ The findings of this study suggest that amoxicillin/clavulanic acid is not only the most frequently prescribed antibiotic in hospital outpatient clinics but also the most common inappropriately prescribed antibiotic for dose guidelines. Resistance of *Klebsiella pneumoniae* and *Escherichia coli* to amoxicillin/clavulanic acid has become a significant and clinically relevant problem due to the extensive use of this antibiotic. Fortunately, limiting the use of amoxicillin/clavulanic acid effectively reduces amoxicillin/clavulanic acid resistance.^36^

In fact, outpatient care is cheaper and safer than hospital admission for children with non-severe pneumonia. The prescribing cost for amoxicillin/clavulanic acid and azithromycin antibiotics was the highest, accounting for 57.15% and 23.00%, respectively due to their high frequency of prescriptions. Besides, the cost of consumption of foreign antibiotics was found to be 9.0 times higher than that of Vietnam antibiotics. The amount and cost of consuming brand-name antibiotics were also found to be 2.0 times higher than that of generic antibiotics if calculated by packaging units. When calculated by dose units, brand-name antibiotics (56.5%, n=13,909) were also prescribed more often than generic antibiotics (46.5%, n=10,729). This may be due to the higher effectiveness of foreign medicine/brand name medicine in pneumonia treatment than Vietnam medicine/generic medicine and the request for better prescriptions containing foreign or brand name medicine from the patient's family. However, it has been established that the risks associated with the use of brand names could increase financial costs for families.^37^ WHO and DOH have encouraged healthcare facilities to start offering more generic medicine options to give patients better value for money. The optimal percentage of drugs prescribed by the generic name of WHO was 100%. Therefore, many countries around the world have followed the WHO and succeeded in encouraging the use of generic medicine over brand-name medicine. For instance, the prescription rates for generic medicine in Germany, the UK, and UAE were 80.0%, 78.0%, and 100.0%, respectively.^38^ whereas the prescription rate of generic medicine in Vietnam was found to be low (about 35%) for outpatient CAP treatment of children in this study.

### Strengths and limitations

The strength of the present study is the observation of a large number of prescriptions over a long period of time (3-years) in a tertiary pediatric hospital, so the study results may be generalizable to the pediatric population with “non-severe” CAP and national representative. In addition, this study used electronic software to generate data related to antibiotic use so as not to miss any prescriptions that met the study criteria.

The limitations of this study are the use of the retrospective method and the lack of data on the clinical outcomes of antibiotic prescribing, which may help better understand the rationality of antibiotic choice decisions. However, the study provides baseline information for comparison with future studies to assess the impact of interventions, to monitor and evaluate prescribing practices.

### Recommendation

Based on the findings of this study, treatment of “non-severe” pneumonia in children should be encouraged to prescribe single antibiotics based on laboratory test results. Therefore, rapid and accurate diagnostic testing for viruses and bacteria should be an important component of a doctor's plan to diagnose any infection. In addition, education about the limited dangers and benefits of antibiotic use needs to be further promoted to reduce antibiotic resistance. The present study also recommends reorienting evidence-based training on rational medicine use for all healthcare professionals along with an annual review of antibiotic guideline compliance in each hospital.

To prevent the indiscriminate use of antibiotics, particularly if the infection is viral, molecular diagnostic tests for the microorganisms *Streptococcus pneumoniae, Haemophilus influenzae* type b, and *Mycoplasma pneumoniae* should be used. As previously said, this would prevent the following: using more antibiotics than prescribed, as this might result in antibiotic resistance, greater side effects, a higher chance of hazardous drug combinations, incorrect medicine administration, non-adherence to prescribed dosages, and increased.

## Conclusions

In summary, this retrospective study presents important long-term data in a tertiary pediatric hospital and provides further insight into the outpatient antibiotic treatment of children with pneumonia in Vietnam. Outpatient CAP prescriptions were found more frequently in males than in females and were more commonly prescribed in the age group 0 to 60 months. The antibiotics amoxicillin/clavulanic acid and azithromycin were most frequently prescribed and generally had good guideline adherence rates, except for high non-adherence to the dose guidelines for amoxicillin. Clinicians generally prefer the combination of amoxicillin/clavulanic acid (penicillin) and azithromycin (macrolide) for the treatment of non-severe pneumonia. The proportion of combination antibiotics was found to be high and showed an increasing trend in the outpatient treatment of non-severe CAP in Vietnam. Because all pediatric patients were not assigned bacterial and viral testing for non-severe CAP, clinicians empirically prescribed antibiotics, which is a known risk factor for drug resistance. Moreover, the low prescription rate of generic antibiotics is the main reason for the increase in the patient's treatment costs. Antibiotic treatment (one and two antibiotics), antibiotic class, and antibiotic origin exhibited significant association with rational and irrational antibiotic prescriptions (dose and dose interval) with p-values<0.05. Similarities with previous studies have emphasized that outpatient antibiotic use for CAP should be examined locally.

## CRediT authorship contribution statement

**Tuong Vi Le Thi:** Methodology, Supervision, Data curation, Investigation, Formal analysis, Resources, Writing – original draft. **Em Canh Pham:** Methodology, Supervision, Data curation, Conceptualization, Investigation, Writing – original draft, Writing – review & editing. **Doan-Trang Dang-Nguyen:** Methodology, Conceptualization, Investigation, Supervision, Writing – review & editing.

## Conflicts of interest

The authors declare that they have no known competing financial interests or personal relationships that could have appeared to influence the work reported in this paper.

## Appendix Supplementary materials

Image, application 1
