# Supplementary material for: Evaluation of children's antibiotics use for outpatient pneumonia treatment in Vietnam
Source: Braz J Infect Dis. 2024 Jul 9;28(4):103839. doi: 10.1016/j.bjid.2024.103839 (PMC11321292; doi:10.1016/j.bjid.2024.103839)

**BJID-D-24-00047_ Supplementary information**

**Supplementary information**

**Table S1** Number of prescriptions (n) for outpatient pneumonia treatment by month.

|  | **Month** | **1** | **2** | **3** | **4** | **5** | **6** | **7** | **8** | **9** | **10** | **11** | **12** | **Total** |
| --- | --- | --- | --- | --- | --- | --- | --- | --- | --- | --- | --- | --- | --- | --- |
| **2019** | n | 132 | 88 | 118 | 158 | 170 | 183 | 217 | 182 | 212 | 245 | 177 | 169 | **2051** |
|  | % | 6.4 | 4.3 | 5.8 | 7.7 | 8.3 | 8.9 | 10.6 | 8.9 | 10.3 | 11.9 | 8.6 | 8.2 | **100** |
|  | * | 1.38 | 1.06 | 0.92 | 1.69 | 1.51 | 1.43 | 1.69 | 1.37 | 1.73 | 1.81 | 1.43 | 1.35 | **1.45^a^** |
| **2020** | n | 135 | 57 | 18 | 8 | 9 | 23 | 32 | 51 | 78 | 285 | 189 | 160 | **1045** |
|  | % | 12.9 | 5.5 | 1.7 | 0.8 | 0.9 | 2.2 | 3.1 | 4.9 | 7.5 | 27.3 | 18.1 | 15.3 | **100** |
|  | * | 1.27 | 0.64 | 0.26 | 0.32 | 0.09 | 0.22 | 0.27 | 0.57 | 0.70 | 1.92 | 1.41 | 1.43 | **0.76^a^** |
| **2021** | n | 140 | 60 | 63 | 82 | 67 | 11 | 3 | 7 | 3 | 6 | 10 | 7 | **459** |
|  | % | 30.5 | 13.1 | 13.7 | 17.9 | 14.6 | 2.4 | 0.7 | 1.5 | 0.7 | 1.3 | 2.2 | 1.5 | **100** |
|  | * | 1.27 | 0.86 | 0.53 | 0.69 | 0.72 | 0.34 | 0.19 | 0.61 | 0.21 | 0.14 | 0.14 | 0.10 | **0.48^a^** |
| **Total** | n | 407 | 205 | 199 | 248 | 246 | 217 | 252 | 240 | 293 | 536 | 376 | 336 | **3555** |
|  | % | **11.4** | 5.8 | 5.6 | 7.0 | 6.9 | 6.1 | 7.1 | 6.8 | 8.2 | **15.1** | **10.6** | **9.5** | **100.0** |
|  | * | 1.31 | 0.85 | 0.57 | 0.90 | 0.77 | 0.66 | 0.72 | 0.85 | 0.88 | 1.29 | 0.99 | 0.96 | **0.90^a^** |

n, Number of prescriptions.

* The proportion of outpatient pneumonia/total outpatient prescriptions (%).

^a^ Mean of 12-months.

**Table S2** Demographic characteristics of children with Community-Acquired Pneumonia (CAP).

| **Year** |  | **n** | **%** | **Other characteristics** |
| --- | --- | --- | --- | --- |
| **2019** | Male | 1099 | **53.6** | n_total_ = 2051 (place of residence: Ho Chi Minh City (51.8%) and other Cities/Provinces (48.2%) with 100% oral route |
|  | Female | 952 | **46.4** |  |
|  | 2 to <60 | 1885 | **91.9** | Mean=28.07 months (max=172.63, min=2.00) |
|  | ≥60 | 166 | **8.1** |  |
| **2020** | Male | 601 | **57.5** | n_total_ = 1045 (place of residence: Ho Chi Minh City (42.2%) and other Cities/Provinces (57.8%)) with 100% oral route |
|  | Female | 444 | **42.5** |  |
|  | 2 to <60 | 986 | **94.4** | Mean=26.62 months (max=170.50, min=2.00) |
|  | ≥60 | 59 | **5.6** |  |
| **2021** | Male | 244 | **53.2** | n_total_ = 459 place of residence: Ho Chi Minh City (38.6%) and other Cities/Provinces (61.4%) with 100% oral route |
|  | Female | 215 | **46.8** |  |
|  | 2 to < 60 | 422 | **91.9** | Mean=28.17 months (max=151.97, min=2.00) |
|  | ≥ 60 | 37 | **8.1** |  |
| **Total** | Male | 1944 | **54.7** | n_total_ = 3555 (place of residence: Ho Chi Minh City (44.17%) and other Cities/Provinces (55.83%) with 100% oral route |
|  | Female | 1611 | **45.3** |  |
|  | 2 to < 60 | 3292 | **92.6** | Mean=27.23 months (max=172.63, min=2.00) |
|  | ≥ 60 | 263 | **7.4** |  |

n, Number of prescriptions; Mean, Mean age of pediatric patients with CAP.

**Table S3** Combination of outpatient antibiotics for CAP treatment in children.

| **Combination of antibiotics** | | **2019** | | **2020** | | **2021** | | **Total** |
| --- | --- | --- | --- | --- | --- | --- | --- | --- |
|  |  | **f** | **%** | **f** | **%** | **f** | **%** | **%** |
| **Amoxicillin/Clavulanic acid** | **Azithromycin** | 499 | **76.07** | 279 | **76.02** | 139 | **77.22** | **76.44** |
| Amoxicillin/Clavulanic acid | Clarithromycin | 8 | 1.22 | 8 | 2.18 | 3 | 1.67 | 1.69 |
| Amoxicillin/Clavulanic acid | Erythromycin | 9 | 1.37 | 3 | 0.82 | 2 | 1.11 | 1.10 |
| Amoxicillin/Sulbactam | Azithromycin | 19 | 2.90 | 2 | 0.54 | 0 | 0.00 | 1.15 |
| Amoxicillin | Azithromycin | 2 | 0.30 | 5 | 1.36 | 2 | 1.11 | 0.92 |
| **Cefuroxime** | **Azithromycin** | 67 | **10.21** | 37 | **10.08** | 11 | **6.11** | **8.80** |
| **Cefpodoxime** | **Azithromycin** | 39 | **5.95** | 26 | **7.08** | 18 | **10.00** | **7.68** |
| Cefpodoxime | Clarithromycin | 2 | 0.30 | 1 | 0.27 | 0 | 0.00 | 0.19 |
| Cefditoren | Azithromycin | 2 | 0.30 | 1 | 0.27 | 2 | 1.11 | 0.56 |
| Cefixime | Azithromycin | 3 | 0.46 | 1 | 0.27 | 0 | 0.00 | 0.24 |
| Cefuroxime | Clarithromycin | 3 | 0.46 | 3 | 0.82 | 1 | 0.56 | 0.61 |
| Cefuroxime | Erythromycin | 3 | 0.46 | 0 | 0.00 | 1 | 0.56 | 0.34 |
| Cefditoren | Clarithromycin | 0 | 0.00 | 1 | 0.27 | 0 | 0.00 | 0.09 |
| Cefdinir | Azithromycin | 0 | 0.00 | 0 | 0.00 | 1 | 0.56 | 0.19 |
| **Total** | | 656 | 100 | 367 | 100 | 180 | 100 | 100 |

f, Frequency (number of times/year); Total, Average of 3-years.

**Table S4** Cost by the origin of antibiotics.

| **Antibiotic group** | **2019** | | | | **2020** | | | **2021** | | **Total** | |
| --- | --- | --- | --- | --- | --- | --- | --- | --- | --- | --- | --- |
|  | **Consumption amount** | | **Consumption cost** | | **Consumption amount** | **Consumption cost** | | **Consumption amount** | **Consumption cost** | **Consumption amount** | **Consumption cost** |
|  | **n** | **%** | **%** | **n** | **%** | **%** | **n** | **%** | **%** | **%** | **%** |
| Vietnam medicine | 3762 | 33.85 | 11.75 | 1692 | 28.86 | 9.66 | 679 | 24.91 | 9.23 | 29.21 | 10.21 |
| Foreign medicine | 7351 | 66.15 | 88.25 | 4170 | 71.14 | 90.34 | 2046 | 75.06 | 90.77 | 70.78 | 89.79 |
| Generic medicine | 4372 | 39.34 | 28.93 | 2049 | 34.95 | 31.55 | 881 | 32.32 | 38.20 | 35.54 | 32.89 |
| Brand-name medicine | 6741 | 60.66 | 71.07 | 3813 | 65.05 | 68.45 | 1845 | 67.68 | 61.80 | 64.46 | 67.11 |
| **Total** | 11113 | 100 | 100 | 5862 | 100 | 100 | 2726 | 100 | 100 | 100 | 100 |

n, Quantity in units of packing; Total, Average of 3-years.

**Table S5** Cost by antibiotic class.

| **Antibiotic** | | **2019** | | **2020** | **2021** | **Total** |
| --- | --- | --- | --- | --- | --- | --- |
| **Group** | **Group** | **%** | | **%** | **%** | **%** |
| Penicillin | Amoxicillin | 0.47 | | 0.64 | 0.39 | 0.50 |
| Penicillin/*β*-lactamase inhibitor | **Amoxicillin/Clavulanic acid** | **58.80** | | **56.34** | **56.32** | **57.15** |
|  | Amoxicillin/Sulbactam | 1.05 | | 0.51 | 0.00 | 0.52 |
| 2^nd^ generation Cephalosporin | **Cefuroxime** | **10.85** | | **9.28** | **5.79** | **8.64** |
|  | Cefaclor | 0.01 | | 0.12 | 0.46 | 0.20 |
| 3^rd^ generation Cephalosporin | Cefdinir | 0.00 | | 0.09 | 0.17 | 0.09 |
|  | **Cefpodoxime** | **3.17** | | **7.06** | **9.03** | **6.42** |
|  | Cefditoren | 0.57 | | 0.94 | 1.43 | 0.98 |
|  | Cefixime | 0.08 | | 0.05 | 0.02 | 0.05 |
| Macrolide | **Azithromycin** | **23.12** | | **21.39** | **24.49** | **23.00** |
|  | Clarithromycin | 1.39 | | 3.41 | 1.76 | 2.19 |
|  | Erythromycin | 0.21 | | 0.17 | 0.15 | 0.18 |
| Fluoroquinolone | Ciprofloxacin | 0.02 | | ‒ | ‒ | 0.02 |
|  | Levofloxacin | 0.003 | | ‒ | ‒ | 0.00 |
| Lincosamide | Clindamycin | 0.27 | | ‒ | ‒ | 0.27 |
|  | | | 100 | 100 | 100 | 100 |

Total, Average of 3-years.

**Figure S1** Comparison of total outpatient prescriptions for CAP treatment in three years by month.


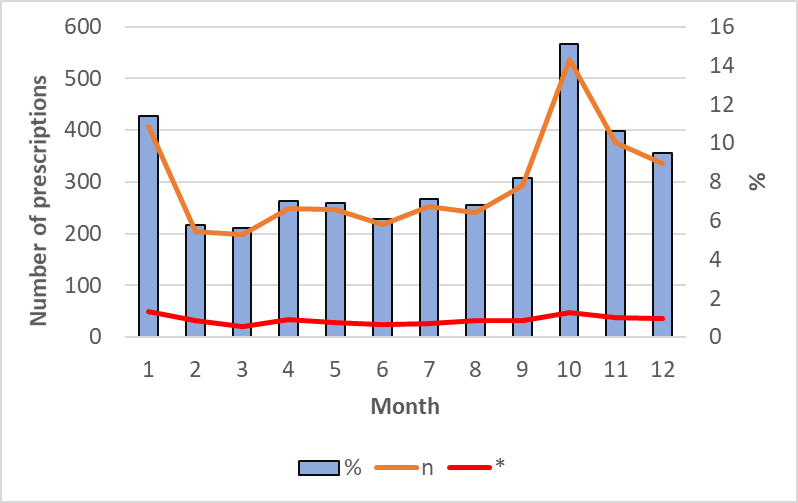


**Figure S2** Compliance and non-compliance with antibiotics prescribed guidelines by prescription unit. (Compliance ‒ agreed on both dose and dose interval guidelines for all antibiotics in each prescription).


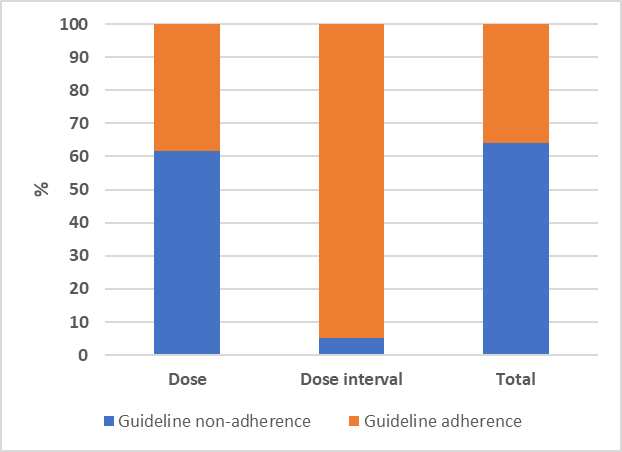

Supplement: Supplementary file 1 [file mmc1.docx]
